# Periparturient Changes in Voluntary Intake, Digestibility, and Performance of Grazing Zebu Beef Cows with or without Protein Supplementation

**DOI:** 10.3390/ani14111710

**Published:** 2024-06-06

**Authors:** Gabriel Santos Souza David, Ellém Maria de Almeida Matos, Bianca Rodrigues Domingos, Yuri Cesconetto Ebani, Luiz Carlos Oliveira de Sousa, Gabriela Duarte Oliveira Leite, Pedro Henrique Borba Pereira, Luciana Navajas Rennó, Sidnei Antônio Lopes, Sebastião de Campos Valadares Filho, Mário Fonseca Paulino

**Affiliations:** 1 Departament of Animal Science, Universidade Estadual Paulista, Jaboticabal 14884-900, SP, Brazil; biancarodrigues.domingos@gmail.com; 2 Departament of Animal Science, Universidade Federal de Viçosa, Viçosa 36570-900, MG, Brazil; ellem.matos@ufv.br (E.M.d.A.M.); yuri.ebani@ufv.br (Y.C.E.); luizcsousa@ufv.br (L.C.O.d.S.); duarte.gaby2010@hotmail.com (G.D.O.L.); pedro.borba.pereira@gmail.com (P.H.B.P.); lucianarenno@ufv.br (L.N.R.); sidnei.lopes@ufv.br (S.A.L.); scvfilho@ufv.br (S.d.C.V.F.); mpaulino@ufv.br (M.F.P.)

**Keywords:** beef cattle, feed intake, pasture

## Abstract

**Simple Summary:**

Several studies with *Bos taurus* cows report a decrease in voluntary intake close to parturition. However, there are few studies on the evaluation of these parameters in grazing Nellore cows receiving protein supplementation, which could mitigate the decrease in forage intake and improve animal performance. Hence, this study sought to understand how the feed intake and performance of Nellore cows on pasture changes during the peripartum period. Our study found a significant reduction in cows’ voluntary intake as they approach parturition, which provides a rational approach to supplementing pregnant cows at the end of gestation, improving production rates in calf–cow systems in the tropics.

**Abstract:**

We aimed to understand the changes in nutritional parameters and performance of beef cows during the peripartum, whether receiving or not receiving protein supplements. Forty cows were used, divided into two treatments: CON—mineral mix and SUP—protein supplementation. Digestibility trial was performed at 45, 30, and 15 days (d) before the parturition and at 20 and 40 d of lactation. The ADG of cows pre- and postpartum was recorded along with the BCS in gestational (GT) and maternal (MT) tissues in the prepartum. There was an effect of treatment and period (*p* ≤ 0.044) for intakes of DM and CP. The forage intake was similar (*p* > 0.90) but varied with the effect of days related to parturition (*p* < 0.001). There was a 14.37% decrease in DM intake from d −30 to d −15 of prepartum. In the postpartum, at 20 d of lactation, there was an increase of 72.7% in relation to d −15 of prepartum. No differences were observed in postpartum ADG or BCS at parturition and postpartum (*p* ≥ 0.12). However, higher total and MT ADG (*p* ≤ 0.02) were observed in animals receiving supplementation, while ADG in GT remained similar (*p* > 0.14). In conclusion, there is a decrease in intake for pregnant cows close to parturition and greater performance of animals supplemented in prepartum.

## 1. Introduction

In tropical conditions, pregnant beef cows are commonly raised on pastures, where they usually experience mid-to-late gestation during the dry season. This environmental scenario often results in nutrition restrictions for the cows [1]. Some studies have demonstrated that protein supplementation during the prepartum (post-weaning) period has a more pronounced effect on animal performance compared to supplementation during other physiological phases of beef cows [2]. However, there is a lack of studies assessing how protein supplementation impacts the dynamics of body tissues in beef cows on pasture during the peripartum period.

Understanding changes in voluntary intake during both late gestation and early lactation is crucial, as these are the periods with the highest nutritional requirements for beef cows [3]. Typically, voluntary intake in *Bos Taurus* females declines nearing parturition [4], possibly due to limited ruminal space caused by the gravid uterus expansion. After parturition, there is an increase in feed intake [5]. In contrast, there is evidence that physical constraints on feed intake during late gestation may be compensated by an increased passage rate [5].

Furthermore, understanding regarding the extent of the decline in feed intake towards the end of gestation in Zebu beef cows under grazing remains to be understood. This could provide a strategic approach for supplementing grazing pregnant beef cows in calf–cow systems in the tropics. Overall, studies investigating the effects of the gestation period on feed intake in beef cows have primarily focused on *Bos Taurus*, which exhibits some physiological differences compared to Zebu cattle [6].

In the tropics, studies have revealed that protein supplementation may improve forage intake by improving the adequacy of substrates (i.e., energy and protein) in both metabolism and the rumen [7]. Therefore, we point out that cows receiving protein supplementation during late gestation might not experience severe restrictions in forage intake, thus potentially improving animal performance.

Our hypothesis is that grazing Nellore beef cows exhibit a decrease in voluntary intake close to parturition, followed by an increase after parturition. However, we anticipate that this decrease will be less pronounced in cows receiving protein supplementation, improving animal performance and maternal tissue loss. Hence, we aimed to investigate the pattern of voluntary intake and digestibility in Zebu beef cows under grazing throughout the peripartum period. Simultaneously, we aimed to understand whether protein supplementation during this period alters the pattern of feed intake and animal performance.

## 2. Materials and Methods

The experiment was conducted at the Beef Cattle Facility of the Animal Science Department at the Universidade Federal de Viçosa, Minas Gerais, Brazil (20°45′ S and 42°52′ W). All animal care and handling procedures were approved by the Animal Care and Use Committee of the Universidade Federal de Viçosa (Protocol 045/2021).

### 2.1. Animal Management, Experimental Design, and Treatments

Forty multiparous Nellore cows carrying male fetuses (F1 Nellore x Red Angus), with initial body weight (BW) of 525 ± 46 and initial body condition score (BCS) of 5.25 ± 0.85, were used. Cows were submitted to a fixed-time artificial insemination protocol using semen from the same sire. Cows (experimental unit) were randomly allocated into eight paddocks with seven hectares each, evenly covered with *Urochloa decumbens* grass, with free access to water and feeders.

The experiment was performed according to a completely randomized design. Two treatments were evaluated: control cows, which received only a mineral mixture throughout the entire experiment, and supplemented cows, which received a daily protein supplement at the amount of 1.0 kg a cow per day. The supplement was provided daily at 11:00 h to minimize any interference with animal grazing behavior. Treatment application started 60 days before parturition (220 days of gestation) and continued until 40 days after parturition. All animals were rotated among the paddocks every 7 days, aiming to control the possible effects of paddocks on treatments.

The supplement was formulated to contain 28% crude protein (CP) as supplied to meet approximately 24% of the CP maintenance requirements for a pregnant cow averaging 525 kg, 235 days of gestation, and expected calf birth weight of 32 kg, according to the Nutrient Requirements of Zebu and Crossbred Cattle-BR-CORTE [8]. The chemical composition of the supplement and pasture can be found in Table 1.

### 2.2. Sample Collection and Measurements

The BCS and BW of the cows were recorded 45 days and 7 days prior to the estimated parturition date, on the day of parturition, and 30 days after parturition. The BCS was assessed by three trained observers using a scale from 1 to 9 [9]. Cows were weighed at 08:00 h, except on the day of parturition. Calves’ weights were recorded at birth and 30 days after birth. The pregnancy rate of cows at the end of the breeding season also was recorded. During the breeding season, the cows were synchronized, and FTAI was performed. The pregnancy diagnosis was conducted by transrectal ultrasonography. The synchronization protocol was performed as follows: an intravaginal device of progesterone release (Tecnopec Primer, São Paulo, Brazil) was introduced, and cows received an injection of 2.0 mg of oestradiol benzoate (Tecnopec Primer, São Paulo, Brazil) on day 0. On day 7, the intravaginal device was removed, and a 2 mL injection of cloprostenol sodium (MSD Saúde Ciosin Animal, São Paulo, Brazil) was administered. On day 8, cows received 0.5 mL of oestradiol cypionate via injection (Zoetis-Pfizer E.C.P., Campinas, Brazil).

Intake and apparent digestibility trials were performed 45, 30, and 15 days before the expected parturition date and 20 and 40 days after parturition, using markers. Each trial lasted nine days, with five days to stabilize the marker’s fecal excretion [10], followed by four days for sample collection. Fecal output was estimated using chromic oxide as an external marker. Chromic oxide was infused via the esophagus at a dose of 15 g per animal from the first to the eighth day of each trial. Individual supplement intake was estimated using titanium dioxide, being mixed daily with the supplement in the amount of 15 g per animal [11]. Forage intake was estimated using indigestible neutral detergent insoluble fiber (iNDF) as an internal [12]. Fecal samples were taken immediately after defecation or directly from the rectum of the animals. Fecal collections were scheduled at 18:00, 14:00, 10:00, and 06:00 h on days 6, 7, 8, and 9 of each trial, respectively [13]. Samples were pooled per animal and period (i.e., 45, 30, and 15 days before the expected parturition date and 20 and 40 days after parturition).

On the last day of each trial, forage samples were taken from each paddock by hand-plucked sampling in order to assess the chemical composition of consumed forage. Concurrently, forage availability was assessed by using a metal square (0.5 × 0.5 m) at four randomly chosen points within each paddock. A second forage sample was pooled per paddock.

### 2.3. Laboratory Analysis and Calculations

Samples of forage, supplement, and feces were oven-dried at 55 °C and subsequently processed to pass through 1 mm and 2 mm sieves. The contents of dry matter (DM; dried for 16 h at 105 °C; INCT method-CA G-003/1), ash (complete combustion at 550 °C; method M-001/2), and N (Kjeldahl method; INCT-CA method N-001/2) were evaluated according to the standard analytical procedures of the Brazilian National Institute of Science and Technology in Animal Science INCT-CA [14] using the samples processed at 1 mm. The content of neutral detergent fiber (NDF) was evaluated according to Mertens et al. [15] using a heat-stable α-amylase and omitting sodium sulfite. The content of NDF was expressed with correction for contaminant ash and protein (NDFap). The content of indigestible neutral detergent fiber (iNDF) was estimated through a 288 h in situ incubation procedure using samples processed at 2 mm [16].

The chromium concentration in the feces samples was assessed through atomic absorption spectrophotometry (GBC Avanta Σ, Scientific Equipment, Braeside, Victoria, Australia) using digestion techniques with nitric and perchloric acids, at the ratio of 3:1 v v^−1^, in one-step digestion with sodium molybdate as catalyst [17]. The concentration of titanium dioxide in the fecal samples was evaluated by spectrophotometry (INCT-CA; method M-007/2).

The average daily gain (ADG) in maternal and gestational tissues was calculated. For this, non-pregnant shrunk body weight (SBWnp), pregnant shrunk body weight (SBWp), and pregnancy components (PREG) were calculated based on the models described by Gionbelli et al. [18], where PREG = gravid uterus plus udder accretion during the pregnancy (GUdp) + weight of udder of a pregnant cow minus the udder weight of the cow in a non-pregnant condition (UDdp); GUdp = increase in the gravid uterus during pregnancy (difference between the weight of the pregnant uterus and the weight of the uterus in the non-pregnant condition) and UDdp = increase in udder weight during pregnancy (difference in weight of the udder of the pregnant cow and the estimated weight of the udder in the non-pregnant condition). The SBW (shrunk body weight) was calculated in the pregnant condition, where SBWp = 0.8084 × BWp^1^.^0303^, where BWp = body weight of the pregnant cow. The SBW of a non-pregnant cow corresponds to the difference between the SBWp and the PREG (SBWnp = SBWp − PREG).

Potentially digestible DM (pdDM) was estimated according to Paulino et al. [18] as follows:

pdDM = 0.98 × (100 − NDF) + (NDF − iNDF)(1)

Fecal output was estimated by the ratio between the amount of chromium supplied and its concentration in the feces. Individual supplement intake (*SI*; kg/day) was estimated through the ratio of titanium dioxide in the feces to the concentration of the indicator in the supplement, as follows:

(2) $$SI=\left[\frac{FO\times CMf}{IS}\right]\times SOG$$

where *FO* is the fecal output (kg/day); *CMf* is the concentration of the marker in the feces (kg/kg); *IS* is the marker present in the supplement offered to each group (kg/day); and *SOG* is the supplement amount offered to the group of animals or treatment (kg/day).

Forage intake (*FI*) was calculated from the following equation:

(3) $$FI=\frac{\left[\left(FO\times iNDFf\right)–\;SIx\times iNDFs\right]}{iNDFfor}$$

where *FO* is the fecal output (kg/day); iNDFf is the concentration of iNDF in the feces (kg/kg); *SI* is the supplement DM intake (kg/day); iNDFs is the concentration of iNDF in the supplement (kg/kg); and iNDFfor is the concentration of iNDF in the forage (kg/kg).

### 2.4. Statistical Analysis

The experiment was analyzed according to the following model:

Y*_ij_* = µ + T*_i_* + e_(*i*)*j*_

where Y*_ij_* is the observation taken in the experimental unit *j* submitted to the treatment *i*; µ is the general constant; T*_i_* is the fixed effect of treatments (control or protein supplement); and e_(*i*)*j*_ is the random error, assumed to be NIID (0, σ_ɛ_^2^).

The cow was considered the experimental unit. Intake and digestibility characteristics were analyzed by considering the day related to parturition as repeated measures. The choice of the best structure of the (co)variance matrix was based on the lowest Akaike’s information criterion value. The degrees of freedom were estimated by the Kenward–Roger method. Effects of maternal treatments, days related to parturition, and interaction between them were analyzed.

Data related to the animal performance were analyzed separately for the pre- and postpartum phases. When pertinent, the initial body weight of the cows was used as a covariate in the model.

Significance was declared at *p* < 0.05, and trends were considered at 0.10 ≥ *p* ≥ 0.05. All statistical analyses were carried out using the PROC MIXED procedure of SAS 9.4 (Inst. Inc., Cary, NC, USA). Binary data (i.e., pregnancy rate) were analyzed using the GLIMIXX procedure of SAS.

## 3. Results

### 3.1. Intake and Apparent Digestibility

On average, the availability (kg/ha) and herbage allowance (kg pdDM/100 kg of body weight) of the *Urochloa decumbens* grass were 3267 and 6.31, respectively.

Overall, protein supplementation increased the intake (*p* ≤ 0.044; Table 2) of DM, OM, CP, and digested organic matter (DOM) but did not affect the intake of NDFap and NDFi (*p* ≥ 0.67). On average, supplemented cows exhibited a 9.13% higher total DM intake compared to the cows receiving only the mineral mixture. No interaction (*p* ≥ 0.16) effect between treatments and days related to parturition was observed on voluntary intake.

**Figure 1:**
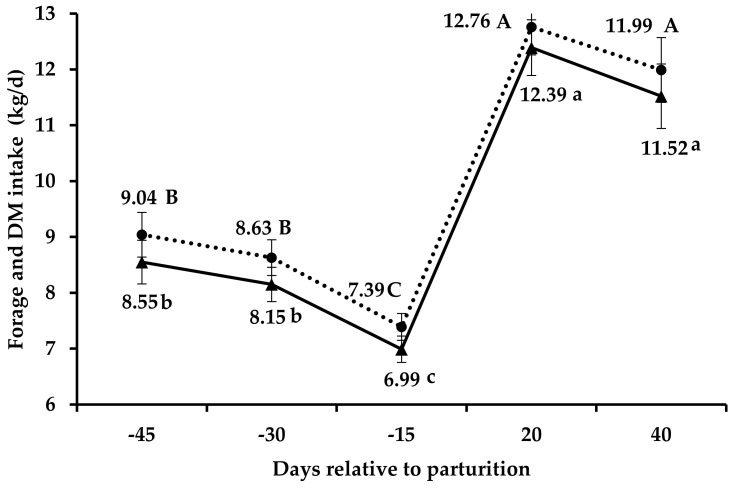
Intake of forage (solid line) and total dry matter (dotted line) in the peripartum grazing Nellore cows. Means followed by different lowercase letters (*p* < 0.001) between forage intake periods are different. Means followed by different capital letters (*p* < 0.001) between periods of total dry matter intake are different.

On the other hand, there was an effect (*p* ≤ 0.001) of days related to parturition for all characteristics of voluntary intake (Table 2). The intake of total DM and forage decreased (*p* < 0.01) by 14.37 and 14.23%, respectively, from the 250th (30 days before parturition) to the 265th day of gestation (15 days before parturition; Figure 1). Likewise, from the end of gestation (15 days before parturition) to the early lactation (20 days after parturition), there was an increase (*p* < 0.01) of 72.7 and 77.3% in total DM and forage intake, respectively (Figure 1).

**Figure 2:**
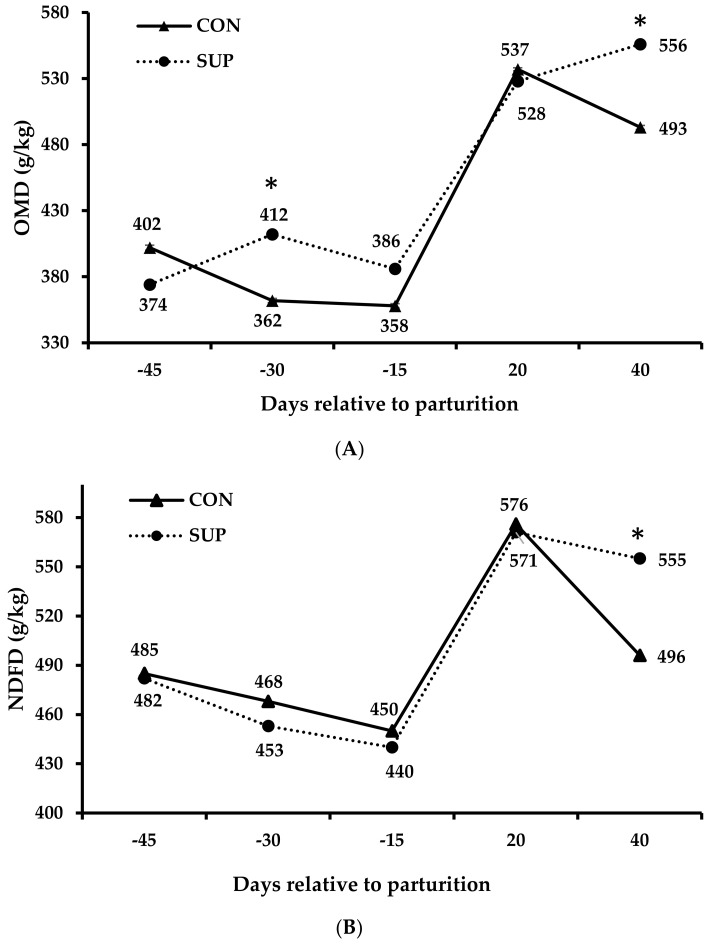

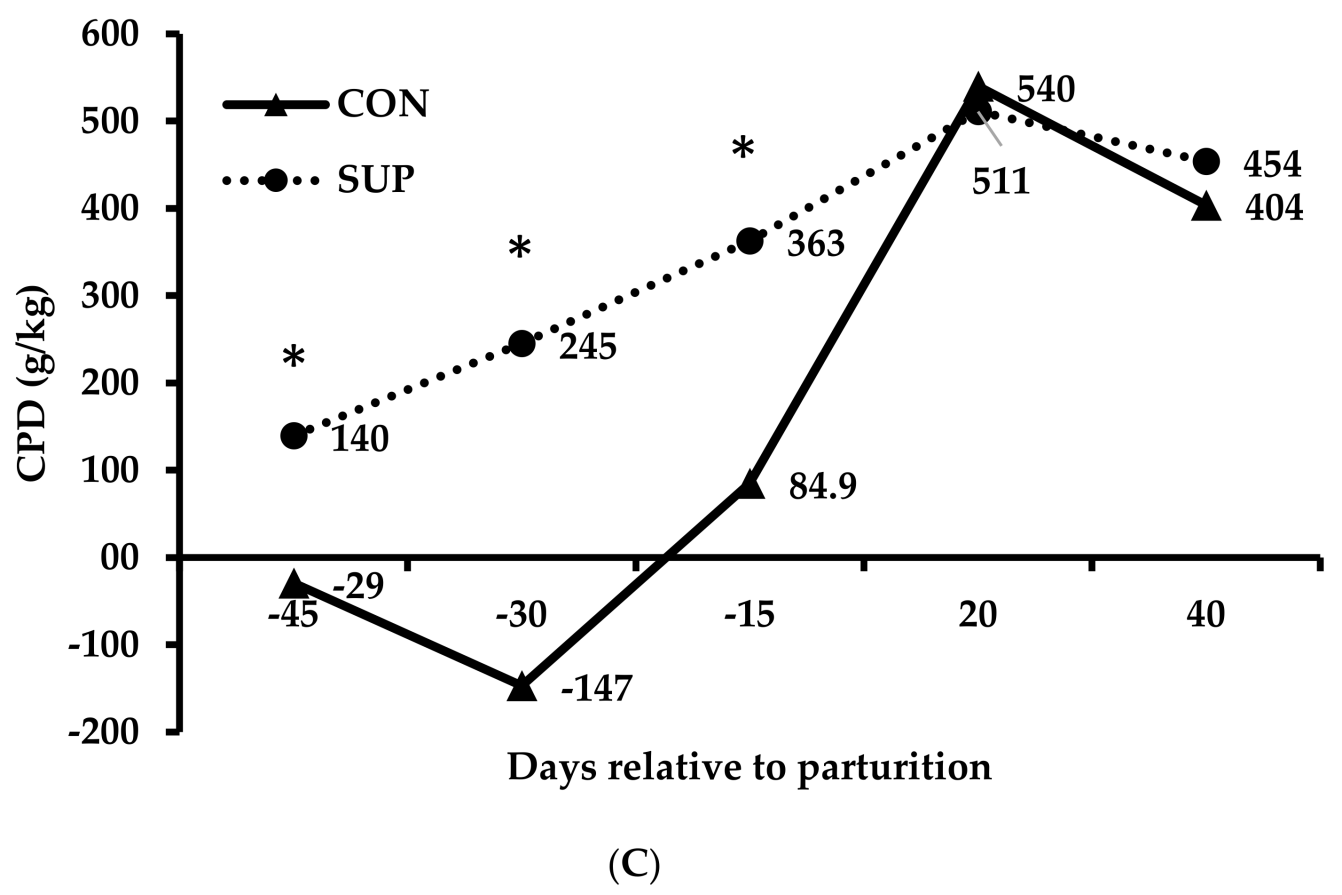
Apparent digestibility of organic matter (**A**), neutral detergent fiber corrected for ash and protein (**B**), and protein (**C**) throughout the peripartum of grazing Nellore cows. The treatment means (CON and SUP) within each period accompanied by * are different from each other (*p* < 0.05).

The crude protein intake remained the same (*p* < 0.05) during the prepartum period, showing an increase 20 days after parturition but with an intermediate value 40 days after parturition (*p* < 0.05). There was a 19% decrease (*p* < 0.05) in NDFap intake from the 250th (30 days before parturition) to the 265th day of gestation (15 days before parturition; Figure 2), with higher intake at 20 days after parturition (*p* < 0.05).

There was an effect (*p* ≤ 0.066) interaction between treatments and days related to parturition for all apparent digestibility characteristics (Table 3). The slicing of this interaction effect showed that protein supplementation increased (*p* ≤ 0.05) OM digestibility at the last 30 days of gestation and at 40 days after parturition, but no difference (*p* ≥ 0.05) was detected among the other days related to parturition (Figure 2A). On the other hand, we observed a higher NDFap digestibility in supplemented cows only 40 days after parturition (*p* < 0.05; Figure 2B). In contrast, protein supplementation improved (*p* ≤ 0.001) CP digestibility during the prepartum phase, whereas no effect (*p* ≥ 0.15) was observed during the postpartum period (Figure 2C).

### 3.2. Performance

**Figure 3:**
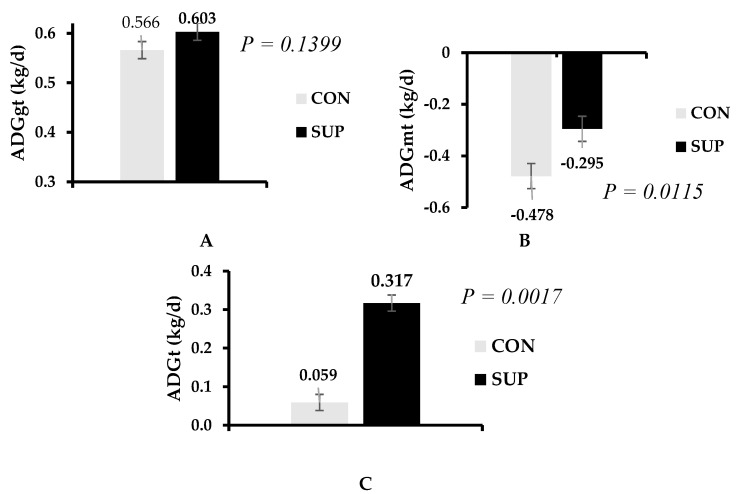
Average daily gain in gestational tissues ((**A**); ADGgt), maternal tissue ((**B**); ADGmt), and total ((**C**); ADGt) during prepartum of grazing Nellore cows receiving or not protein supplementation.

Protein supplementation increased (*p* ≤ 0.012) both total and maternal ADG in the prepartum period (Figure 3A,B). Conversely, there was no effect of treatments (*p* > 0.13) on gestational ADG (Figure 3C). Similarly, protein supplementation did not affect (*p* ≥ 0.12) BSC at parturition and lactation, postpartum ADG, pregnancy rate, and calves’ weights at birth and at the age of 30 days (Table 4).

## 4. Discussion

The pdDM constitutes an integrative measure of both quantitative and qualitative characteristics of the forage as it simultaneously defines the available forage that is potentially convertible into animal products. In tropical regions, some authors have suggested that a minimum of 4 to 5 kg of pdDM/100 kg of BW should be ensured to allow selective grazing by animals and, therefore, not affect voluntary intake and performance [19]. It is noteworthy that throughout all experiments, the herbage allowance (kg pdDM/100 kg BW) remained stable and within the recommended range. Thus, any effects of inadequate pdDM availability on animal performance would be unlikely.

In this study, cows showed body weight loss in maternal tissues during late gestation. Indeed, several authors have reported a pattern of transition from the anabolic state to the catabolic state in pregnant beef cows, on average, from 240 days of gestation [20]. During late gestation, there is an increase in the cow’s protein requirements [3]. In our study, forage had low CP content during the dry season (on average, 57 g CP/kg DM). Thus, it is reasonable to state that cows increased body mobilization as an attempt to meet the demands for both fetal-placental growth and development, as well as meet the N demands for microbial growth in the rumen via recycling [21,22].

Even though all cows lost maternal tissue, protein supplementation avoided the mobilization from the cows’ body reserves, leading to higher total weight gain. This can be explained, at least partially, by the additional supply of nutrients, especially protein, via protein supplementation. In agreement, protein supplementation has been reported to increase the mRNA expression of skeletal protein synthesis markers in supplemented cows [23]. Furthermore, additional N supply has been reported to enhance N balance, reducing reliance on skeletal muscle as a source of amino acids in pregnant heifers. This has been accompanied by a decreased abundance of proteins related to muscle degradation [24].

Otherwise, protein supplementation did not affect gestational tissue gain, suggesting that the non-supplemented cows adjusted their metabolism to avoid nutrient deficiencies for the fetus. During gestation, females from all species undergo homeorhesis. Thus, mammal females tend to prioritize fetal growth, exhibiting coordinated changes in their tissue metabolism to regulate nutrient partitioning needed to support the fetus [25,26]. Additionally, it has been demonstrated that under a metabolizable protein deficiency, placental blood flow is increased, indicating an adaptation of the placental vasculature [27]. In these lines, the placenta may enhance the abundance of glucose transporter 3 (GLUT-3) in an attempt to increase its capacity for placental glucose transfer [28].

Despite some studies reporting a higher calf birth weight in cows supplemented during late gestation [29], the pattern of metabolism adjustment (i.e., homeorhesis) corroborates with similar calf birth weights between treatments. This result aligns with some of the studies in tropical conditions, which demonstrate the lack of effects of maternal supplementation for beef cows during late gestation on calf birth weight [30,31]. The variation in responses among the studies can be attributed to the level of restriction experienced by non-supplemented cows (i.e., pasture quality), as well as the amount of supplement offered to supplemented cows.

Early lactation is a critical period, as the peak of lactation occurs from 3 to 5 weeks after parturition in Nellore cows [32]. This period is typically followed by numerous metabolic, physiological, and hormonal changes, all occurring in an integrated manner to support the demands of nutrients for milk synthesis [25]. In our study, peripartum protein supplementation was unable to avoid postpartum weight loss. It has been reported that postpartum protein supplementation improves milk yield [33] but does not affect productive performance [31]. Our data showed that maternal protein supplementation did not affect offspring performance at the age of 30 days. This result may suggest that milk yield was not affected by treatments.

In cow–calf operations, feed supplementation should be utilized at specific times when the efficiency of supplement utilization by beef cows is optimized [34]. In the tropics, late gestation in beef cows usually aligns with the dry season. In this regard, substantial evidence suggests that feed supplementation of beef cows during late gestation is more effective than during early lactation [2,3,4,5,6,7,8,9,10,11,12,13,14,15,16,17,18,19,20,21,22,23,24,25,26,27,28,29,30].

Assessing BCS at late gestation and at parturition is essential as it accurately estimates body energy reserves in beef cows, allowing predictions of reproductive success and understanding the impacts of nutritional strategies on animal performance [35]. It is noteworthy that cows in both treatments presented adequate body condition (averaging 5.0) at parturition [36]. This can be partially attributed to the adequate herbage forage allowance. Under these conditions, differences in pregnancy rate would not be expected.

Providing adequate N supply in the rumen is crucial for optimizing the digestion of fibrous compounds and increasing forage intake [37]. Furthermore, maximizing forage intake is related to the metabolic adequacy of absorbed nutrients [38]. Our hypothesis posited that protein supplementation could avoid the decrease in the cow’s feed intake close to parturition as it potentially enhances fiber degradation, leading to an increased passage rate and thereby promoting increased forage intake. However, we observed that the decrease in feed intake as pregnancy progressed was similar in both treatments.

Findings from other studies suggest that forage utilization would be optimized when dietary CP is raised to 100 g/kg DM by protein supplementation [39]. In our study, supplementation increased dietary CP close to 70 g/kg DM during the dry season. This could explain the lack of effect of protein supplementation on forage intake during the dry season. In contrast, during the rainy season, forage itself already had a CP content close to 100 g/kg DM, which is considered an optimized diet for the use of pasture. Thus, we would not expect any effect of protein supplementation on forage intake during the rainy season.

Our primary concern was to understand changes in feed intake throughout the peripartum period. We would expect a decrease in voluntary intake close to parturition, which was confirmed in both treatments. Exponential fetal growth reduces gastrointestinal capacity, leading to decreased voluntary intake [20,40,41]. Moreover, it is important to note that in ruminants, approximately 75% of fetal growth takes place during late gestation, which further imposes constraints on rumen capacity [42]. Therefore, cows may be unable to meet their nutrient requirements, leading to maternal weight loss, as observed in our study. Indeed, we observed an average decrease of 14% in forage intake from the 250 to the 265th day of gestation. This finding aligns with studies conducted with *Bos taurus* pregnant beef heifers, wherein a 9.1% decrease in voluntary intake was reported in the last week of gestation [5].

In the tropics, several authors have reported that forage qualitative characteristics such as CP and iNDF contents are closely associated with forage intake in grazing cattle [43]. It is worth mentioning that even with an improvement in pasture quality during late gestation (i.e., a decrease in indigestible fiber and an increase in CP), cows drastically reduced their feed intake. This observation confirms that the compression of the uterus in the rumen prevails over any other effect on intake.

After parturition, there was a 77% increase in forage intake compared to the last evaluation period during pregnancy. Lactating ruminants have a higher intake compared to non-lactating ruminants. In fact, differences of up to 100% in voluntary intake have been observed for pregnant or lactating sheep and cattle. However, some authors suggest that constraints on cows’ intake capacity persist during the first week of lactation. This constraint arises as the rumen is still returning to its normal volume, with increased intake from this stage onwards [44]. In fact, some studies have observed a decrease in rumen weight at the end of pregnancy, followed by a re-establishment of the cows’ intake capacity 20 days after parturition [45,46].

As expected, the intakes of DM, DOM, and NDFap followed the variations observed in forage intake throughout the periods. Conversely, variations in CP intake did not align with the forage intake pattern. This discrepancy could be attributed to the highest CP content in prepartum forage during the period of lowest intake (i.e., 15 days before the parturition). This finding justifies the similarity in CP intake by animals throughout the prepartum period. The increase in OM digestibility for animals supplemented at 40 days of lactation is associated with greater NDFap digestibility in this period. Additionally, the enhanced CP digestibility for supplemented cows is explained by the greater CP intake, which increases its participation in the total diet, reducing the relative participation of the metabolic fecal fraction [47]. The higher total DM intake in supplemented cows is explained exclusively by the supplement intake, as there was no effect of treatments on forage intake. The similar NDFap intake between treatments reflects the lack of effect of treatments on forage intake, as forage represents most of the dietary fiber.

This study brings a rational, practical approach to supplementation strategies for grazing *Bos indicus* beef cows, as it shows a marked decrease in voluntary intake at the end of gestation. While protein supplementation may not improve the nutritional characteristics and birth weight of calves, it effectively mitigates the loss of maternal tissue. 

## 5. Conclusions

Regardless of protein supplementation, grazing Zebu beef cows exhibit a decline in voluntary intake as parturition approaches, followed by a subsequent increase postpartum. While protein supplementation may not improve the nutritional characteristics, it effectively mitigates the loss of maternal tissue. This aspect is crucial for achieving favorable results in cow–calf operations.

## Figures and Tables

**Table 1:** Chemical composition of the supplemented and pasture, and forage mass.

Item	Supplement ^1^	Forage ^2^				
		−40	−30	−15	20	40
*Chemical composition*						
Dry matter (as-fed)	890	450	453	370	273	301
Organic matter, g/kg DM	972	901	910	911	916	910
Crude protein, g/kg DM	288	48	50	72	102	92
NDFap ^3^, g/kg DM	86	696	674	643	553	548
iNDF ^4^, g/kg DM	9	253	262	247	169	171
NDIN ^5^, g/kg total nitrogen	23	229	233	304	363	384
*Forage availability*						
Forage mass (kg/ha)	-	3429	3302	3097	3070	3440
Herbage allowance	-	6.57	6.24	5.92	6.00	6.93

^1^ Soybean meal (100 g/kg); corn meal (760 g/kg); urea (60 g/kg); and mineral mix (80 g/kg): CaHPO_4_ = 500 g/kg; NaCl = 476.25 g/kg; ZnSO4 = 15 g/kg; Cu_2_SO_4_ = 7.5 g/kg; CoSO_4_ = 0.5 g/kg; KIO_3_ = 0.5 g/kg; MnSO_4_ = 0.25 g/kg. ^2^ Days relative to parturition. ^3^ Neutral detergent fiber with correction for contaminant ash and protein. ^4^ Indigestible neutral detergent fiber. ^5^ Neutral detergent insoluble nitrogen.

**Table 2:** Effects of protein supplementation on voluntary intake of grazing beef cows during the peripartum period.

Item ^1^	Treatments		SEM ^2^	*p*-Value ^3^		
	CON	SUP		SUP	Period	S × P
*Intake (kg/d)*						
Dry matter	9.53	10.40	0.28	0.04	<0.001	0.96
Forage DM	9.53	9.50	0.27	0.93	<0.001	0.91
Organic matter	8.69	9.50	0.25	0.03	<0.001	0.99
DOM	3.90	4.69	0.14	0.02	<0.001	0.95
Crude protein	0.72	0.93	0.02	<0.001	<0.001	0.16
NDFap	5.81	5.71	0.14	0.67	<0.001	0.94
iNDF	1.97	1.95	0.05	0.80	<0.001	0.64
*Intake, g/kg BW*						
Dry matter	19.0	20.4	0.05	0.10	<0.001	0.91
Forage DM	19.1	18.6	0.05	0.55	<0.001	0.78
Crude protein	17.3	18.6	0.05	0.07	<0.001	0.96
NDFap	11.5	11.1	0.02	0.33	<0.001	0.96
iNDF	3.88	3.83	0.01	0.76	0.002	0.49

^1^ DOM = dietary content of digested organic matter; NDFap = neutral detergent fiber corrected for contaminants ash and protein. ^2^ SEM = standard error of the mean. ^3^ Sup. = effect of protein supplementation; S × P = interaction between protein supplementation and days relative to parturition (P).

**Table 3:** Effects of protein supplementation on apparent digestibility (g/kg) of grazing beef cows during peripartum period.

Item ^1^	Treatments		SEM ^2^	*p*-Value ^3^		
	CON	SUP		SUP	Period	S × P
Organic matter	430	451	0.81	0.09	<0.001	0.06
NDFap	495	500	0.76	0.64	<0.001	0.09
Crude protein	160	334	1.58	<0.001	<0.001	<0.001

^1^ NDFap = neutral detergent fiber corrected for contaminants ash and protein. ^2^ SEM = standard error of the mean. ^3^ Sup. = effect of protein supplementation; S × P = interaction between protein supplementation and days relative to parturition (P).

**Table 4:** Effects of protein supplementation on performance of grazing beef cows during peripartum period.

Item ^1^	Treatments		SEM ^2^	*p*-Value
	CON	SUP		
Postpartum ADG	−0.11	−0.13	0.14	0.93
Parturition BCS	5.37	5.11	0.12	0.13
Postpartum BCS	4.87	4.96	0.16	0.47
Calf birth weight	32.5	34.4	0.87	0.12
CBW30	69.8	72.3	2.53	0.47
Pregnancy rate (%)	80	73.7	-	0.64

^1^ CBW30 = calf weight at the age of 30 days; ^2^ SEM = standard error of the mean.

## Data Availability

The data presented in this study are available upon request from the corresponding author.
